# *Candida auris* in South Africa, 2012–2016

**DOI:** 10.3201/eid2411.180368

**Published:** 2018-11

**Authors:** Nelesh P. Govender, Rindidzani E. Magobo, Ruth Mpembe, Mabatho Mhlanga, Phelly Matlapeng, Craig Corcoran, Chetna Govind, Warren Lowman, Marthinus Senekal, Juno Thomas

**Affiliations:** National Institute for Communicable Diseases, a division of the National Health Laboratory Service, Johannesburg, South Africa (N.P. Govender, R.E. Magobo, R. Mpembe, M. Mhlanga, P. Matlapeng, J. Thomas);; University of the Witwatersrand, Johannesburg (N.P. Govender, R.E. Magobo, W. Lowman);; Ampath Laboratories, Pretoria, South Africa (C. Corcoran);; Lancet Laboratories, Durban, South Africa (C. Govind);; Vermaak and Partners Pathologists, Johannesburg/Cape Town, South Africa (W. Lowman):; Pathcare Pathologists, Johannesburg (M. Senekal)

**Keywords:** *Candida auris*, yeast, *Candida*, laboratory surveillance, fungi, South Africa

## Abstract

To determine the epidemiology of *Candida auris* in South Africa, we reviewed data from public- and private-sector diagnostic laboratories that reported confirmed and probable cases of invasive disease and colonization for October 2012–November 2016. We defined a case as a first isolation of *C. auris* from any specimen from a person of any age admitted to any healthcare facility in South Africa. We defined probable cases as cases where the diagnostic laboratory had used a nonconfirmatory biochemical identification method and *C. haemulonii* was cultured. We analyzed 1,692 cases; 93% were from private-sector healthcare facilities, and 92% of cases from known locations were from Gauteng Province. Of cases with available data, 29% were invasive infections. The number of cases increased from 18 (October 2012–November 2013) to 861 (October 2015–November 2016). Our results show a large increase in *C. auris* cases during the study period, centered on private hospitals in Gauteng Province.

The earliest reported case of infection with the yeast *Candida auris* in South Africa occurred in 2009; however, the pathogen was initially misidentified as *Candida haemulonii* (a closely related yeast), and *C. auris* was only confirmed retrospectively in 2014, when 4 other cases of *C. auris* candidemia were described in South Africa (*1*). Since descriptions in Southeast Asia in 2009, cases of *C. auris* have been reported from many countries on 6 continents (Asia, Africa, South America, Europe, North America, and most recently Oceania) (*2*)*.*

*C. auris* has been associated with large healthcare-associated outbreaks because of its ability to be transmitted person-to-person by direct contact, form biofilms, persist in the hospital environment on surfaces and on shared equipment, and resist chemical disinfection by certain products (*3**–**5*)*.* Over the past 9 years, cases of *C. auris* have been detected at many hospitals in South Africa, causing large outbreaks at some facilities, and this pathogen now accounts for ≈1 of every 10 cases of candidemia (*6*)*.* South Africa has a unique *C. auris* clade separated from Asian, Southeast Asian, and South American clades by tens of thousands of single-nucleotide polymorphisms, consistent with the hypothesis that *C. auris* emerged independently in Africa and simultaneously on several other continents (*7*). However, the prevalence and geographic extent of *C. auris* disease is likely underestimated, especially in low- and middle-income countries in Africa, because conventional laboratory methods misidentify the fungus and relatively few resource-limited countries have the capacity for identification by mass spectrometric or molecular methods (*8*)*.* South Africa has an established national surveillance infrastructure for infectious diseases, including those caused by antimicrobial drug–resistant pathogens, that is based on a large network of well-equipped diagnostic pathology laboratories. In light of an emerging epidemic of *C. auris* infections among hospitalized patients in parts of South Africa, we sought to describe the national epidemiology of laboratory-confirmed cases during 2012–2016.

## Materials and Methods

We conducted national laboratory-based surveillance for *C. auris* retrospectively over a period of >4 years, from the earliest known reports of cases in South Africa in October 2012 through November 2016 (*1*)*.* We defined a case as a first isolation of *C. auris* from any specimen from a patient of any age admitted to any South Africa healthcare facility. We also included probable cases in which the diagnostic laboratory had used a nonconfirmatory biochemical identification method such as Vitek-2 YST (bioMérieux, Marcy ľEtoile, France) and *C. haemulonii* was cultured. The National Health Laboratory Service (NHLS) provides diagnostic pathology services to the public sector, serving ≈83% of the population of South Africa, and has ≈60 mostly hospital-based laboratories offering tests for fungal identification. NHLS laboratories performed species-level identification for *Candida* NHLS laboratories using several platforms during the surveillance period, namely Vitek 2 YST, API 20C Aux, or API ID 32C (bioMérieux); Auxacolor (Bio-Rad, Hercules, CA, USA); and Microscan (Beckman Coulter, Brea, CA, USA). Some of these diagnostic platforms are known to misidentify *C. auris* as other yeasts (*9*)*;* however, we did not include any other species in our probable case definition. Private pathology laboratory practices, which serve the remainder of the population with health insurance, have a centralized model of fungal identification; that is, central laboratories perform diagnostic tests for patient specimens referred from a large number of healthcare facilities across a region or province. These laboratories used the same yeast identification platforms; however, 1 private pathology practice introduced the Vitek MS system (bioMérieux) in 2013. In general, NHLS laboratories identified *Candida* to species level only for isolates from normally sterile sites, whereas private laboratories identified all *Candida* isolates to species level, regardless of the specimen source. 

We obtained line list specimen-level data from 4 large private diagnostic pathology practices, which together serve almost the entire private health sector, for the surveillance period. We requested that these line lists included any specimens from which either *C. haemulonii* or *C. auris* was cultured. We deduplicated these laboratory data to patient level by applying the surveillance case definition and using a unique laboratory identifier. We then merged this dataset with a similar line list of cases of *C. auris* fungemia that were submitted to the National Institute for Communicable Diseases (NICD) from NHLS laboratories as part of candidemia surveillance. All these bloodstream isolates were confirmed as *C. auris* at NICD using the Bruker Biotyper system (Bruker, Bremen, Germany) or PCR amplification and sequencing of the internal transcribed spacer (ITS) domain of the ribosomal RNA gene using universal primers (*10*). Variables included in the final dataset were age or date of birth, sex, date of specimen collection, location (province and admitting hospital), specimen type, and species-level identification. 

We defined a case as colonization if the isolate was cultured from central venous catheter tips (with no corresponding blood culture specimen), urine, respiratory tract specimens and skin or mucosal swabs. We defined a case as invasive disease if the source of the isolate was blood, cerebrospinal fluid, or serous fluid or tissue. Treating clinicians or hospital-based infection prevention and control (IPC) practitioners submitted the specimens that yielded these isolates; therefore, we may have detected cases of colonization because of active screening at some hospitals with outbreaks. There was no uniform practice for screening for colonization during the surveillance period, and this study preceded the interim guidance issued by NICD (*11*). To our knowledge, the number of healthcare facilities served by the laboratory network did not change over the surveillance period. We obtained approval for laboratory-based surveillance from the Human Research Ethics Committee (Medical), University of the Witwatersrand, Johannesburg.

## Results

For October 2012—November 2016, we identified a total of 1,692 confirmed or probable *C. auris* cases at both public and private hospitals, although most patients (1,578/1,692, 93%) were admitted to private facilities. Of the private-sector cases, at least 647 (38%) had isolates that were identified as *C. haemulonii* and not confirmed as *C. auris*. All 114 bloodstream isolates from public-sector cases were confirmed as *C. auris* at NICD’s mycology reference laboratory. Of 1,579 case-patients with a recorded specimen type, 451 (29%) had invasive disease (Table), with isolates cultured from normally sterile sites: 344 (76%) from blood, 56 (12%) from fluid, 49 (11%) from tissue, and 2 (<1%) from cerebrospinal fluid. The remaining 1,128/1,579 (71%) isolates were cultured from sites of probable colonization: 622 (55%) from urine, 288 (26%) from central venous catheter tips, 173 (15%) from respiratory tract, and 45 (4%) from skin, mucosal, or wound swabs. 

**Table:** Characteristics of cases of *Candida auris* invasive disease versus colonization, South Africa, 2012–2016*

Characteristic	Cases with available data, n = 1,579	Invasive disease, n = 451	Colonization, n = 1,128
Median patient age, y (interquartile range)†	n = 1,576	55 (41–68)	63 (49–74)
Patient sex, no. (%)	n = 1,540	n = 442	n = 1,098
M	957 (62)	273 (62)	684 (62)
F	583 (38)	169 (38)	414 (38)
Health sector, no. (%)	n = 1,549	n = 439	n = 1,110
Private	1,435 (93)	325 (74)	1,110 (100)
Public	114 (7)	114 (26)	0
Province, no. (%)	n = 1,465	n = 424	n = 1,041
Gauteng	1,336 (91)	380 (90)	956 (92)
Mpumalanga	72 (5)	25 (6)	47 (5)
North West	20 (1)	7 (2)	13 (1)
Other provinces	37 (3)	12 (2)	25 (2)

*Specimen type data were unavailable for 116 cases. †Compared using a Wilcoxon rank-sum test.

Male patients accounted for 62% of cases. The median age of patients was 60 years (IQR 46–72 years); 38 patients (2%) were <18 years of age, and 9 (0.5%) were infants <1 year of age. Patients with invasive disease were younger than colonized patients: median age for invasive disease was 55 years (IQR 41–68 years) versus median 63 years (IQR 49–74 years) for colonized patients (p <0.001). All cases of colonization were detected at private-sector hospitals; at least 39% (444/1,128) of these isolates had been identified as *C. haemulonii*. In contrast, 25% (112/451) of invasive isolates had been identified as *C. haemulonii.*

**Figure 1:**
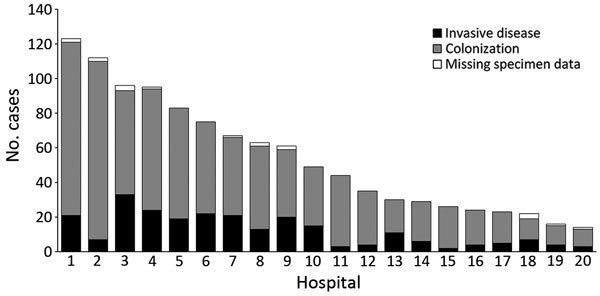
Distribution of cases of *Candida auris* by type of infection, South Africa, 2012–2016. Data are from the top 20 private hospitals that reported cases. n = 1,087.

**Figure 2:**
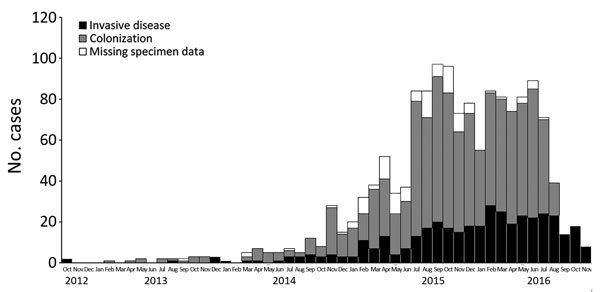
Distribution of cases of *Candida auris* by type of infection and date of specimen collection, South Africa, 2012–2016. n = 1,306.

Of cases with a known location, 92% (1,440/1,571) were reported from Gauteng Province, the most densely populated province and the economic and travel hub of South Africa. A median of 4 cases (interquartile range [IQR] 1–11) was reported from each of >94 hospitals. Of all cases with a known location, 80% (1,087/1,353) were reported from 20 private-sector hospitals, 17 of which were located in Gauteng Province (Figure 1). More than 80 cases were detected at each of 4 private hospitals in Pretoria (Gauteng Province). Large outbreaks (>75 cases) also occurred in a private hospital in Johannesburg (Gauteng Province) and another in Rustenburg (North West Province). The number of cases increased dramatically from 18 in the baseline period (October 2012–November 2013) to 861 in a later corresponding period (October 2015—November 2016) (Figure 2). 

## Discussion

We have demonstrated a dramatic increase in the number of confirmed or probable cases of *C. auris* over 4 years in South Africa. Of concern, *C. auris* was detected at a large number of hospitals; most patients were admitted to private-sector hospitals in Gauteng Province. Most cases were probably caused by colonization, but *C. auris* is now also a common cause of invasive disease, accounting for 10% of all cases of candidemia in recent national surveillance (*6*)*.* We collected minimal patient information through this surveillance but were able to document that most cases occurred among adults rather than children. 

The factors that have led to the emergence and rapid spread of a unique clade of *C. auris* in hospitals in South Africa are not well established. Azole-resistant *C. parapsilosis* is already endemic in private-sector hospitals in Gauteng Province (*12*)*.* We have previously hypothesized that these strains of azole-resistant *C. parapsilosis* initially emerged as a consequence of indiscriminate use of fluconazole for prophylaxis and treatment and that suboptimal adherence to IPC practices caused the transmission of the pathogen within hospitals. This setting is the same one in which *C. auris* has become endemic and has caused large hospital outbreaks. 

The trend we observed is consistent with increased detection of *C. auris* in other regions of the world. For instance, India has seen a large increase in the number of *C. auris* cases, from 12 in 2013 to >350 in 2017 (*13*)*.* By the end of May 2018, the United States had 311 cases and 29 probable cases of *C. auris* (*14*)*.*

Although *C. auris* has now been isolated from >94 hospitals across South Africa, most cases were detected at a small number of hospitals in a restricted geographic region. Focused attention on antifungal stewardship and multimodal IPC interventions at these facilities could limit further outbreaks and minimize transmission of this pathogen within South Africa and across South Africa’s borders. Cross-border transmission from South Africa has already been documented (*15*). However, *C. auris* is notoriously difficult to eradicate from hospitals or units once it has become endemic, in part because of its ability to adhere to polymeric surfaces and form biofilms (*16*)*.* Thorough decontamination has been recommended to reduce the environmental bioburden (*2*)*.*

Early detection of *Candida* species is recommended to facilitate appropriate treatment and implement IPC measures; however, standard biochemical platforms cannot reliably identify *C. auris* in microbiology laboratories. Many diagnostic pathology laboratories in South Africa used methods that could not reliably identify *C. auris* during the surveillance period, but these methods have been largely replaced in 2018 with more accurate methods of identification, including Vitek 2 YST-ID with version 8.01 software (bioMérieux), matrix-assisted laser desorption/ionization-time of flight mass spectrometry or internal transcribed spacer, and D1/D2 sequencing (*9*)*.*

Our study provides a national picture of the emergence of *C. auris* in public and private hospitals. However, the surveillance was limited in several respects. We may have underestimated the number of cases in the public sector because NHLS laboratories did not routinely identify isolates from nonsterile sites to species level; even so, the geographic distribution of cases in the private sector is likely to be accurate. The 4 large private pathology practices that contributed data to the study have a combined national coverage of the private health sector. In addition, we have observed a similar geographic distribution of cases of *C. auris* candidemia detected through our national active population-based surveillance (*6*)*.* We believe that we have observed a real increase in cases over time; this finding is particularly true for private laboratories where *Candida* was routinely identified to species level even from nonsterile sites across the surveillance period. However, some laboratories may have changed their species-level identification practices in response to the emergence of *C. auris*. We included *C. haemulonii* in the case definition because many laboratories used the Vitek 2 YST system without a software update at the time of surveillance, but we did not include yeasts for which other diagnostic platforms can mistake *C. auris*. In addition, cases were diagnosed at some but not all facilities on the basis of clinicians submitting appropriate specimens for fungal culture or of IPC practitioners performing active screening for colonization. We extracted routine laboratory data, often with missing data elements, from several sources and applied a specific surveillance case definition. Deduplication of data to patient level may have been flawed because unique identifiers are not universally used in the healthcare system.

In conclusion, we report a large increase in cases of *C. auris* invasive disease and colonization since the first isolation of this yeast in South Africa in 2009. The increase is mostly attributable to cases in private hospitals in Gauteng Province.
